# Caesarean Section is associated with reduced perinatal cytokine response, increased risk of bacterial colonization in the airway, and infantile wheezing

**DOI:** 10.1038/s41598-017-07894-2

**Published:** 2017-08-22

**Authors:** Sui-Ling Liao, Ming-Han Tsai, Tsung-Chieh Yao, Man-Chin Hua, Kuo-Wei Yeh, Chih-Yung Chiu, Kuan-Wen Su, Shih-Yin Huang, Chuan-Chi Kao, Shen-Hao Lai, Jing-Long Huang

**Affiliations:** 1 Community Medicine Research Center, Chang Gung Memorial Hospital at Keelung, Keelung, Taiwan; 2 Department of Pediatrics, Chang Gung Memorial Hospital at Keelung, Keelung, Taiwan; 3 Division of Allergy, Asthma, and Rheumatology, Department of Pediatrics, Chang Gung Memorial Hospital and Chang Gung University, College of Medicine, Taoyuan, Taiwan; 4 Department of Obstetrics and Gynecology, Chang Gung Memorial Hospital at Keelung, Keelung, Taiwan; 5 Division of Pulmonology, Department of Pediatric, Chang Gung Memorial Hospital and Chang Gung University, College of Medicine, Taoyuan, Taiwan

## Abstract

The relationship between cesarean section (CS) and allergic disorders such as asthma and wheezing has been inconsistent, and the mechanisms for their connection remained largely unknown. We aimed to investigate whether CS is associated with infantile wheeze and to explore the connection between CS and several risk factors known to correlate with allergy development. Mononuclear cells were isolated from cord blood and assessed for cytokine responses by ELISA. Bacteria from nasopharyngeal specimens were identified with traditional culture methods. Infant lung function tests were performed at 6 and 12 months of age. IgE levels and clinical outcomes were assessed at 12 months. The result showed that children delivered by CS were associated with increased risk of wheezing (aHR 1.63; 95% CI: 1.01–2.62) and decreased compliance of the respiratory system at 12 months (p = 0.045). In addition, CS was associated with reduced TLR1–2- triggered TNF-α and IL-6 responses at birth. By12 months of age, children delivered by CS had significantly less airway bacterial clearance. Our findings suggested that CS was associated with decreased pro-inflammatory cytokine response to TLR1–2 stimulation, followed by higher abundance of bacterial colonization in the airway during late infancy, thus increasing the risk of infantile wheezing.

## Introduction

The rate of cesarean deliveries has increased considerably in the last decade; thus, the impact of delivery mode on the offspring’s’ future health deserves in-depth investigation. Although numerous studies have shown cesarean section (CS) to be associated with increased risk of developing future asthma and other wheezing disorder^1–6^, several reports found no significant correlation^7–9^. Thus, the first aim of this study was to investigate whether CS is associated with increased risk of infantile wheezing and/or other allergic disorders in a prospective birth cohort study.

Asthma and allergic disorders are currently among the most common chronic diseases in the pediatric population. A number of biologically plausible mechanisms have been suggested to explain the effect of early- life event on subsequent development of asthma. First, altered perinatal TLR response and aberrant changes in cord blood cytokine response such as IL-6, Il-8, IL-10, IL-13, TNF-α and interferon –γ were shown to be related to future asthma^10–13^. Second, numerous recent reports have suggested the role of microbial exposure in allergy development. Results have shown that neonates with bacterial colonization of the airway are at increased risk for developing recurrent wheeze and asthma in early life^14, 15^. Finally, atopic sensitization or IgE elevation are regarded as useful predictors and biomarkers of allergy tendencies^16, 17^. Given the apparent importance of these factors that might have critical influence on the ultimate pattern of allergy development, we aimed to investigate whether delivery by CS has potential effects on these important elements known to associate with infantile allergic disorder.

## Results

### Subject and demographic data

**Figure 1:**
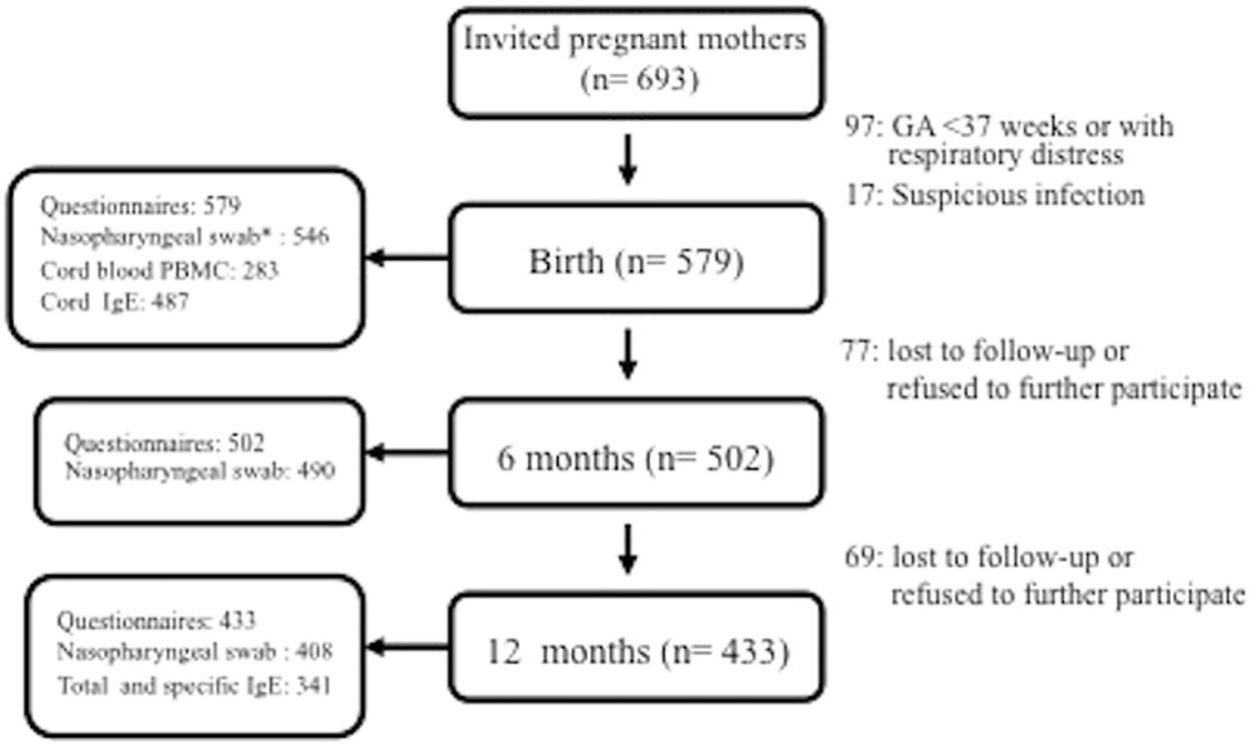
Flow chart of the birth cohort study: demonstrating the number of participants with detailed quantities of various samples tested and valid questionnaire information. ^*^Nasopharyngeal swab was performed at 1 month of age.

Of the 693 invited pregnant mothers, 114 neonates were excluded from this study due to prematurity, respiratory distress, complicated delivery, or were suspicious of infection (detailed exclusion criteria described in the section of Methods). Thus, a total of 579 participants were included in this analysis. The number of participants at each age point and details of performed examinations are listed in Fig. 1. We had excluded premature infants from this analysis because compared to full-term infants, premature infants were known to have more complicated respiratory conditions and distinct innate cytokine response^18^. Baseline characteristics of the infants are shown in Table 1. The rate of CS delivery was 36% in this study. There were no significant differences in the maternal or neonatal characteristics between children born by CS or vaginal delivery, except that children delivered by CS had slightly lower gestational age; however, mean birth body weight remained similar between these 2 groups.

**Table 1:** Demographic characteristic.

Characteristics	NSD n (%)	C/S n (%)	*p*
Sex (male)	199 (53.8)	116 (55.5)	0.95
Gestational Age (week)	38.8 ± 1	38.4 ± 1	<0.01
Birth Body Weight (g)	3154 ± 365	3135 ± 512	0.64
Cord IgE > 0.5 IU/L*	97 (30.7)	57 (33.3)	0.62
House pet	92 (24.9)	57 (27.3)	0.6
Father Allergy	143 (38.6)	77 (36.8)	0.67
Maternal smoking during pregnancy	11 (3.0)	11 (5.3)	0.25
Maternal Allergy	115 (31.0)	79 (37.8)	0.12
Maternal Education			0.66
Primary or secondary	12 (3.2)	7 (3.3)	
High school	89 (24.0)	60 (28.7)	
College or above	269 (72.7)	142 (61.3)	

Total number of NSD: 370.

Total number of C/S: 209.

*Cord IgE: based on 487 samples (NSD: 316; C/S: 171).

### Mode of delivery, clinical outcome, and respiratory mechanics

By the age of one year, 433 infants had their medical records reviewed and completed the questionnaires administered at 6 and 12 months of age. Analysis was made to investigate whether mode of delivery was associated with wheezing, rhinitis, and eczema. The results, summarized in Table 2, showed that CS was associated with increased risk of wheezing by 1 year of age (adjusted HR 1.63; 95% CI: 1.01–2.62). However, mode of delivery was not associated with rhinitis or eczema at any age after adjusting for potential confounding factors.

**Table 2:** Association between mode of delivery and allergic disorders during infancy

	Univariate analysis			Multivariate analysis		
	β	H HR (95%CI)	p	β	HR (95%CI)	p
***6m/o***						
Wheezing	0.31	1.34 (0.90–2.10)	0.15	0.34	1.40 (0.84–2.33)	0.20
Rhinitis	0.25	1.23 (0.91–1.82)	0.15	0.16	1.17 (0.81–1.69)	0.40
Eczema	−0.23	0.80 (0.57–1.12)	0.19	−0.24	0.78 (0.51–1.21)	0.27
***1y/o***						
Wheezing	0.48	1.62 (1.10–2.39)	0.02	0.49	1.63 (1.01–2.62)	0.04
Rhinitis	0.20	1.22 (0.90–1.65)	0.19	0.20	1.22 (0.83–1.82)	0.32
Eczema	0.18	1.21 (0.86–1.70)	0.28	0.35	1.42 (0.93–2.19)	0.11

***6m/o***: 6 months old ***1y/o:*** 1 year old

Adjusted for gestational age, sex, birth body weight, house pet, smoking during pregnancy, breastfeeding duration, and parental allergy

**Figure 2:**
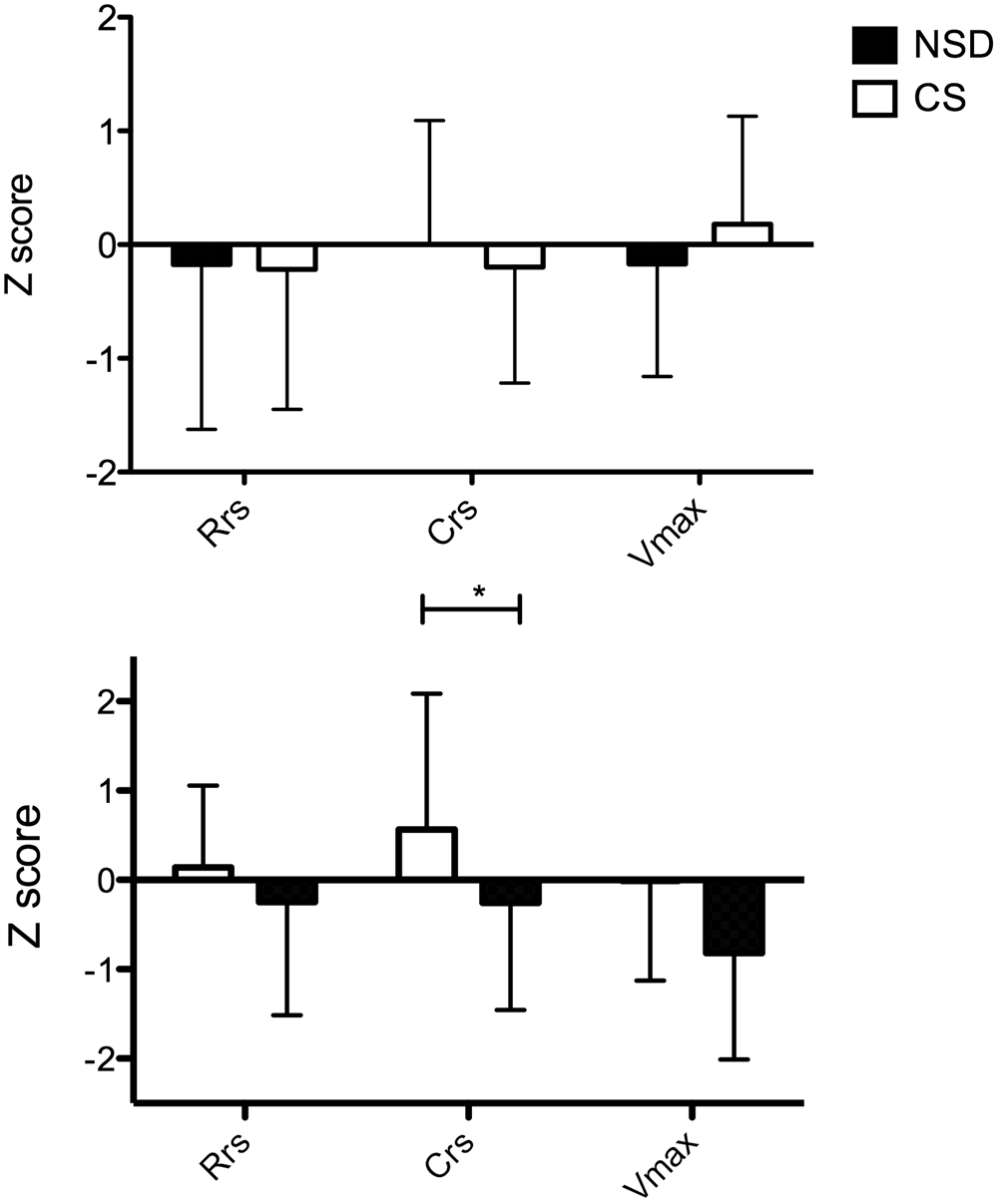
Infant lung function tests were performed in infants at 6 months (upper panel) and 12 months of age (lower panel). Parameters (Rrs: resistance of the respiratory system; Crs: compliance of the respiratory system; and Vmax: maximal expiratory flow at functional residual capacity) were converted into Z score and compared between children delivered vaginally or by cesarean section.

To further support the association between CS and infant wheezing, we had analyzed the relationship between mode of delivery and infant lung function measurements at the age of 6 and 12 months. After adjusting for body length and comparing with local references (values converted into Z scores), infants delivered by CS had significantly lower compliance of the respiratory system (Crs) at the age of 1 year (p = 0.045). However, no differences were noted in the resistance of the respiratory system (Rrs) and maximal expiratory flow at functional residual capacity (Vmax_FRC_) (Fig. 2).

### Mode of delivery and immune biomarkers

**Figure 3:**
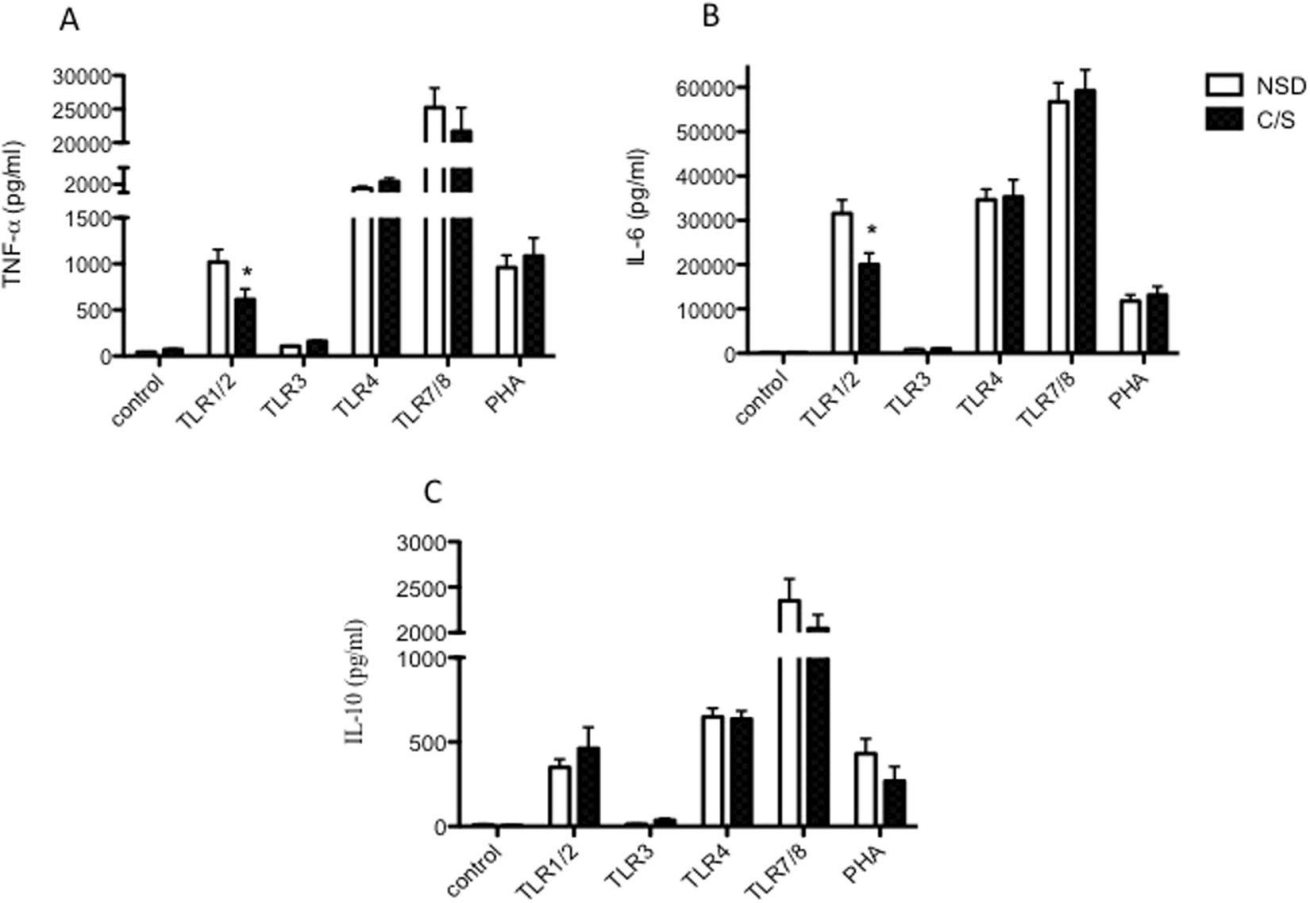
Cord mononuclear cells were collected within few hours of birth and treated with TLR ligands as described in the methods. Supernatants were collected for the analysis of cytokines TNF-α IL-6 (**B**), and IL-10 (**C**) production after stimulation with TLR 1–4, 7/8, and PHA. The results are expressed by means ± SD. Comparisons were made between children born by either delivery mode, and statistical significance determined by student T test (*p-value < 0.05).

To investigate whether the association between CS and wheezing disorder was related to aberrant innate immune function at birth, neonatal cytokine responses to TLR stimulation were compared in children of either delivery method. The results showed that neonates delivered by CS displayed reduced TNF - α and IL-6 response to TLR1–2 stimulation when compared to those born by vaginal delivery. The analysis remained significant after adjusting for confounding factors (p = 0.016 for TNF-α and p = 0.026 for IL-6). No difference was noted for any of the IL-10 responses (Fig. 3).

Next, we investigated whether the association between CS and wheezing disorder was related to atopic sensitization. The result showed that CS was not associated with atopic sensitization or changes in IgE level, both at birth and by 1 year of age (Table 3).

**Table 3:** Association between mode of delivery and IgE level at birth, and total/specific IgE level at 1 year of age.

	Univariate analysisβ (95% CI)	p	Multivariate analysis β (95% CI)	p
Cord blood IgE	0.03 (−0.06–0.11)	0.55	0.04 (−0.22–0.29)	0.78
Total IgE at 1 year	0.23 (0.00–0.46)	0.05	0.22 (−0.02–0.47)	0.07
Specific IgE at 1 year	0.16 (−0.15–0.47)	0.32	0.19 (−0.14–0.52)	0.18

Adjusted for gestational age, parity, gender, birth body weight, smoking during pregnancy, maternal education, and parental allergy.

Cuff-off values:

Cord IgE: ≥0.5 IU/L.

Total IgE: ≥97 IU/L.

Detectable allergen- specific IgE level: ≥0.35 IU/L.

### Mode of delivery and bacterial colonization of the airway

Bacterial colonization of the airway has been shown to increase the risk of recurrent wheezing and asthma in early life^14, 19^. We had then proceeded to investigate whether mode of delivery would modify nasopharyngeal bacterial colonization rate in infants during the first year of life. Assays were performed to identify bacterial species from the nasopharynx as described in the Methods at the age of 1, 6, and 12 months. The results indicated that there was a high but similar bacterial colonization rate in the airway of children delivered by either method at 1 month of age (57% in vaginal and 52% in CS delivered children). Although not significant, bacterial colonization rate appeared slightly higher in the CS group by 6 months of age. Finally, by 12 months of age, bacterial colonization rate in the airway remained significantly higher in CS delivered children (adjusted OR: 1.89; 95% CI: 1.11–3.20). As seen in Table 4, by the end of 1 year, almost ¼ of the children delivered by CS were still colonized with identified bacteria, while only 15% of the vaginally delivered children remained colonized. Methicillin-resistant Staphylococcus aureus accounted for the most common flora identified, followed by Moraxella catarrhalis, and Streptococcus pneumoniae (Supplement). Overall, the result indicated that compared to infants born by vaginal delivery, those children delivered by CS showed slower clearance of bacteria in the airway throughout the first year of life. It was worth mentioning that even though CS was associated with increased bacteria colonization of the airway, it was not associated with increased risk of lower respiratory tract infections, such as bronchiolitis, pneumonia, or croup during infancy (adjusted OR: 0.48; 95% CI: 0.22–1.01 at 6 months and adjusted OR: 0.67; 95% CI: 0.35–1.28 at 12 months of age) (data not shown).

**Table 4:** Effect of mode of delivery on bacterial colonization of the airway at different age point

Age	NSD n (%)	C/S n (%)	Crude OR (95% CI)	p	Adjusted OR (95% CI)	p
1 m/o	196 (57)	106 (52)	0.82 (0.06–1.16)	0.26	0.83 (0.58–1.18)	0.29
6 m/o	71 (23)	55 (30)	1.44 (0.96–2.18)	0.14	1.41 (0.91–2.16)	0.12
12 m/o	39 (15)	34 (23)	1.74 (1.04–2.90)	0.04	1.89 (1.11–3.20)	0.02

Number of samples at 1 month of age (1 m/o): 546 (NSD: 343, C/S: 203).

Number of samples at 6 month of age (6 m/o): 490 (NSD: 308, C/S: 182).

Number of samples at 12 month of age (12 m/o): 408 (NSD: 262, C/S: 146).

Adjusted for gestational age, sex, birth body weight, maternal education, smoking during pregnancy, and parental allergy.

## Discussion

Results from present study showed that CS was associated with increased risk of infantile wheezing and decreased compliance of the respiratory system (Crs). Furthermore, our data had proposed that the association was related to decreased perinatal pro-inflammatory cytokine response to TLR 1–2 stimulation that resulted in higher persistence of bacterial colonization in the airway during late infancy. Although the association between Cesarean birth and infantile wheezing/asthma has remained controversial, the reduced compliance in the lung function test in CS delivered infants had lent some credence to support the connection between cesarean delivery and wheezing disorder, since airway diameter and mechanics (compliance) are important determinants of wheezing and/or flow limitation^20^. In addition, the effects were further evidenced by the relationship between Cesarean birth and aberrant perinatal innate immunity and an increased bacterial colonization of the airway, as both were known to result in future asthma or wheezing disorder^11–15^.

Stress from labor promotes the activity of various cytokines, thus, alteration of this natural process might result in long-lasting effects on the developing immune system. In conformance to several reports that suggested lack of normal labor to result in decreased pro-inflammatory cytokine production, our study had found reduced TNF-α and IL-6 response toward TLR1–2 stimulation in CS delivered neonates^21–23^. Kristensen *et al*. had shown that children delivered by CS are at increased risk of diseases associated with immune function, such as asthma, ulcerative colitis, celiac disease, and lower respiratory tract infections^1^. The relationship between altered innate immune function and allergic disorder was proven by the study of Gold *et al*. that demonstrated reduced cytokine response toward TLR stimulation in children with allergic background^24^. Additionally, monocytes from allergic children showed a significantly lower up –regulation of TLR2 upon stimulation^25^. Collectively, these findings suggested that innate cytokine response might be influenced by Cesarean section, and the consequence of this disturbed immune function might place the children at greater risk of developing immune diseases such as wheezing or allergic disorders.

A number of reports have shown that neonates born by vaginal delivery had increased intestinal bacterial colonization and growth that subsequently primes the developing immune system. However, despite many studies that investigated the relationship between mode of delivery and bacterial colonization in the digestive system, very little is known about the effect of labor on colonization of the upper airway, which poses direct impact on the respiratory health. We are among the first few studies to show the effect of cesarean section on airway colonization. Although our result showed limited direct impact of mode of delivery on nasopharyngeal microbial composition directly after birth, we had observed significant disparities in the trajectory of respiratory microbial development between children born by CS and vaginal delivery over time. Similar to our results, Bosch *et al*. had demonstrated that CS delivered children stayed longer in the S. aureus - dominated profile, therefore, had a higher abundance of pathogenic bacteria compared to vaginally born children at the age of 6 months^26^. Bacterial colonization of the airways with the pathogenic bacteria might stimulate topical inflammatory mediator release, thus, results in subsequent development of asthma or wheezing disorder in early childhood^14, 19, 27^. This outcome can be explained by the reduction in the colonization of health-associated commensals due to lack of exposure to vaginal bacteria during birth, which led to the outgrowth of pathogenic species that affects respiratory health later in life. In addition to this theory, we had found decreased pro-inflammatory cytokine response to TLR1–2 stimulation in the cord blood of CS delivered neonates. Since recognition of Gram- positive bacteria, such as S. aureus and S. pneumoniae occurs via TLR2 pathway (with TLR1 as an essential co-receptor), we speculated that this particular decrease in cytokine response might also be responsible for the slower clearance of bacteria in the CS delivered infants overtime. However, further investigations will be needed to prove this observation. In addition, although bacteria such as group B streptococcus and E. coli are among the most common bacteria isolated in neonates with respiratory diseases, we did not identify such organisms in our population. The main reason might be that infants who harbor such organism usually develop symptoms such as respiratory distress or invasive diseases, thus, had been excluded initially from this study.

Our study has several limitations. First, we have not performed clinical assessment of allergy and nasopharyngeal examination in children beyond 12 months of age. Thus, we were unable to verify if the effect of CS on wheezing disorder or airway bacterial colonization extended beyond infancy. Second, our population size was not big enough to distinguish between emergent and elective CS. The scenarios for emergent CS included prolonged or failed labor induction, fetal distress, uterine rupture, placenta problems, or failed instrumental delivery, etc. All those neonates with clinical distress or premature rupture of membrane were initially excluded from our study. Thus, the majority of the participants in our study had scheduled CS with no risk of infection or complicated deliveries that might impact perinatal immune response. Third, the diagnosis of asthma and wheezing is difficult to ascertain at such young age, thus, one may question the accuracy of the disease outcome. However, besides from information obtained from standard questionnaires, the infants were checked at least once by a study physician, thus, decreasing the possibility of misclassification of wheezing disorder. In addition, it was not likely for the physicians to diagnose wheezing disorder base on the child’s mode of delivery, therefore we believe there was no concern for possible bias. Furthermore, despite a significant association between CS and wheezing disorder, we did not find correlation between CS and atopic sensitization. One possible explanation is that our study participants might have been too young to yet become sensitized. We had previously shown that besides from cow’s milk protein and egg, children were rarely sensitized by other allergen before the age of 3 years^28^. Moreover, although various reports have shown association between CS and wheezing and/or asthma, rarely did they report its correlation with atopic sensitization. Besides, wheezing in infants might sometimes have nothing to do with allergy. Thus, whether if CS has an effect on allergic sensitization would require further investigation. Nevertheless, the lack of association between CS and allergic sensitization might support our theory that the reason for CS associated infantile wheezing might be related to decreased perinatal cytokine response to TLR1–2 that resulted in abundance of bacterial colonization of the airway causing local inflammation, thus, increasing the risk of wheezing disorder.

## Conclusion

To date, despite a wide variety of studies that suggested an association between CS and asthma/wheezing, the underlying mechanism for its connection remained largely unknown. Thus, our results were able to provide some empirical evidence and hypothesis to clarify the myth between CS and future asthma. Our findings suggested that delivery by CS leads to abnormal neonatal innate immune responses (decreased production of TNF-α and IL-6 to TLR1–2 stimulation) and higher abundance of bacterial colonization of the airway in late infancy. Both factors were known to precede the development of wheezing disorder in early life. In addition, our findings implied that the potential mechanism by which CS is associated with higher persistence of airway bacterial colonization in late infancy might partly be related to decreased pro-inflammatory cytokine response to TLR1–2 stimulation since birth. Wheezing disorder constitutes an important disease burden in infants and their parents, and as the rate of CS continues to rise, we believe it is important to further explore the potential mechanisms on how CS can impact the respiratory health in young children.

## Patients and Methods

### Study Population

Data for this analysis came from an ongoing prospective birth cohort study called the PATCH (The Prediction of Allergy in Taiwanese Children). The study was approved by the Chang Gung Ethics Committee (Institutional Review board of Chang Gung Memorial Hospital: IRB reference number 104–6801 and103–6585A3), and all methods performed in the research were in accordance with the relevant guidelines and regulations. Written informed consent was obtained from the parents/legal guardians of the neonates. Pregnant women undergoing routine prenatal exam were invited randomly by a study nurse to join our research program. All neonates were enrolled upon agreement. To exclude potential effects on cytokine response and disease outcome, neonates with the following disorders were excluded from this study: premature delivery (<gestational age of 37 weeks), had major congenital anomaly (include those with underlying parenchyma lung disease), suspicious of congenital infections (such as maternal/neonatal fever, chorioamnionitis, or premature rupture of membrane), those with respiratory distress (such as transient tachypnea of newborn, congenital pneumonia, respiratory distress syndrome, or meconium aspiration syndrome), or 5 minute Apgar score <8. Results from this analysis comprised of the first 579 eligible healthy full-term neonates. Questionnaire survey was conducted at birth to obtain parental information such as demographic characteristics, medical and obstetric history, smoking exposure history, and others. Standardized questionnaires on atopic heredity, environmental factors and presence of atopic symptoms were answered at 1, 6, and 12 months. Clinical diagnosis of allergy or wheezing disorder was made based on questionnaires validated from modified ISSAC questionnaires and physicians’ assessment. By the time of analysis, 433 children included in this study were at least 1 year of age and had adequate follow-up data.

#### Sample collection, cell culture, and TLR ligands stimulation

The details of our experimental procedures have been published previously^29^. Briefly, umbilical cord blood was collected at the time of delivery and mononuclear cells were isolated and stimulated with TLR ligands (TLR 1–4 and 7/8). Cells were treated with NF-kB activator phytohemagglutinin (Murex Pharmaceuticals) at 4 ug/ml in R10-FBS as positive controls. To determine TLR responses, medium or ligands (in duplicate) were added to each well containing 3 × 10^5^ CBMCs in 100 ul R10-FBS and incubated at 37 °C for 20 h with 5% CO2. All assay preparations were performed using sterile technique in a laminar flow hood. The concentrations of the ligands used for this experiment are as follow: 10ug/ml of PAM3csk4 as TLR1/2 ligand, 10ug/ml of poly (I:C) as TLR3, and 20ng/ml of LPS to activate TLR4 pathway, and 10ug/ml of R848 as TLR7/8 ligands (InvivoGen, San Diego, CA).

#### Measurement of cytokines

TNF-α, IL-6, and IL-10 levels in culture supernatants were determined by enzyme-linked immunosorbent assays according to the manufacturer’s instructions (ELISA; R&D systems, MN). The detection limits for TNF-α were 15.6 pg/mL, 3.12 pg/mL for IL-6, and 7.8 pg/mL for IL-10.

#### Bacterial identification from the nasopharyx

Nasopharyngeal specimens were obtained through the nose with cotton-tipped swabs at the age of 1, 6, and 12 months (Copan Swab Applicator, Copan Diagnostics Inc., Brescia, Italy). Specimens were sent to our central microbiology laboratory for standard bacterial cultures within 2 hours of collection. In this study, throat swab were performed only in children free of any respiratory symptoms for at least 3 weeks. Pathogens such as Coag (-) staphylococcus, α, γ –Streptococcus, corynebacterium, Moraxella, etc. were considered as normal flora or low virulence pathogens, thus rarely be considered as pathogenic in the absence of clinical symptoms. Pathogens such as S. pneumoniae, S aureus, H. influenza, Acinetobacter, etc. are considered as potential threat and thus might need colony count to determine their virulence. However, in our study, we looked at colonization (carrier) state and not disease rate, thus, children were considered colonized in the presence of bacteria despite low (rare) growth. Details of our experimental procedures have been published previously^30^.

#### Total and allergen-specific serum immunoglobulin E

Total IgE level was determined by ImmunoCAP (Phadia, Uppsala, Sweden) at birth and by age of 1 year. Specific IgE was determined by a commercial assay for IgE (ImmunoCAP Phadiatop Infant; Phadia) against the most common inhalant and food allergens (i.e. house dust mite, cat, dog, birch, timothy, ragweed, wall pellitory, egg white, cow’s milk, peanut and shrimp). Atopy was defined as having detectable allergen- specific IgE level of ≥0.35 PAU/L. The cuff-off value for cord serum IgE was set at 0.5 IU/L and total serum IgE at 1 year of age was at 97 IU/L^31^.

#### Infant Lung Function Tests

Detailed procedures and data collection of tidal breathing analysis, respiratory mechanics, and forced tidal expiration were described in our previous study^32^. Briefly, Infant lung function testing was performed with the Jaeger Masterscreen BabyBody Paediatrics System (CareFusion, Höchberg, Germany), in conformance with the American Thoracic Society and European Respiratory Society recommendations^20, 33, 34^. Measurements were performed under sedation with oral chloral hydrate (50–75 mg/kg) in healthy infants who had no respiratory tract infection for at least 3 weeks. Children were placed in supine position with neck mildly extended. The oxygen saturation and heart rate of the participants were monitored during the procedure and until they were fully awake. The resistance and compliance of the respiratory system (Rrs and Crs) in respiratory mechanics, and the maximal expiratory flow at functional residual capacity (Vmax_FRC_) in forced tidal expiration were collected for analysis.

### Statistical Methods

We assessed the association between mode of delivery and clinical outcomes (infant wheezing, rhinitis, or eczema) at ages 6 and 12 months by using Cox proportional hazard model. Association between nasopharyngeal bacterial colonization and delivery mode was analyzed by using regression analysis. Neonatal characteristics (birth body weight, gestational age, and gender) and parental history of allergy, smoking during pregnancy, and house pet were included in the multivariate analysis to compensate confounders’ effects. Student T test was used to compare perinatal TLR-induced cytokine level and infant lung function tests (Z score) between infants born by CS or vaginal delivery. A *p*-value of <0.05 was considered significant. All statistical analysis was carried out using IBM SPSS Statistics Version 20 (Armonk, NY).

## Electronic supplementary material

Supplementary Info 1
