# Supplementary material for: Caesarean Section is associated with reduced perinatal cytokine response, increased risk of bacterial colonization in the airway, and infantile wheezing
Source: Sci Rep. 2017 Aug 22;7:9053. doi: 10.1038/s41598-017-07894-2 (PMC5567317; doi:10.1038/s41598-017-07894-2)
Supplement: Supplementary file 1 — Supplementary Info 1 [file 41598_2017_7894_MOESM1_ESM.doc]

Caesarean Section is associated with reduced perinatal cytokine response, increased risk of bacterial colonization in the airway, and infantile wheezing

Sui-Ling Liao^1,2^, Ming-Han Tsai^1,2^, Tsung-Chieh Yao^1,3^, Man-Chin Hua^1,2^, Kuo-Wei Yeh^1,3^, Chih-Yung Chiu^1,2^, Kuan –Wen Su^1,2^, , Shih-Yin Huang^1,4^, Chuan-Chi Kao^1,4^, Shen-Hao Lai^1,5^*, and Jing-Long Huang^1,3^*

Supplement. Detailed bacteria isolates at different age point

| Age | S. aureus  n (%) | S. pneumoniae  n (%) | M. catarrhalis  n (%) | H. influenza  n (%) | Others  n (%) |
| --- | --- | --- | --- | --- | --- |
|  |  |  |  |  |  |
| ***1 m/o***  NSD  CS | 192 (56.0)  102 (50.2) | 1 (0.3)  1 (0.3) | 2 (0.6)  2 (0.6) | 1 (0.3)  1 (0.3) | 0  0 |
| ***6 m/o***  NSD  CS | 57 (18.6)  46 (25.2) | 5 (1.6)  1 (0.5) | 6 (1.9)  4 (2.2) | 3 (1.0)  4 (2.2) | 0  0 |
| ***12 m/o***  NSD  CS | 15 (5.7)  22 (15) | 8 (3.1)  4 (2.7) | 8 (3.1)  4 (2.7) | 7 (2.7)  4 (2.7) | 1 (0.4)  0 |

Others: Acinetobacter sp.

Number of samples at 1 m/o: 546 (NSD: 343, C/S: 203)

Number of samples at 6 m/o: 490 (NSD: 308, C/S: 182)

Number of samples at 12 m/o: 408 (NSD: 262, C/S: 146)
